# Comorbidities and impact on quality of life in patients with Ménière’s disease: a single-center cross-sectional study

**DOI:** 10.3389/fneur.2026.1855669

**Published:** 2026-06-24

**Authors:** Zhongbao Zhang, Linna Jiao, Guohua Zhang, Qingfeng Rong, Qiuli Wang, Xiaohui Lu, Qin Wang

**Affiliations:** 1 Xiaolan Clinical Institute of Shantou University Medical College, Zhongshan, Guangdong Province, China; 2 Department of otolaryngology,Xiaolan People’s Hospital of Zhongshan (The Fifth People’s Hospital of Zhongshan), Zhongshan, Guangdong Province, China; 3 Department of Neurology, Xiaolan People’s Hospital of Zhongshan (The Fifth People’s Hospital of Zhongshan), Zhongshan, Guangdong Province, China; 4 Dizziness and Vertigo Treatment Center,Xiaolan People’s Hospital of Zhongshan (The Fifth People’s Hospital of Zhongshan), Zhongshan, Guangdong Province, China

**Keywords:** anxiety, comorbidity, hypertension, MDoQ, Ménière’s disease, quality of life

## Abstract

**Background:**

Ménière’s disease (MD) is a chronic vestibular disorder that significantly impacts patients’ quality of life (QoL). However, the influence of comorbid conditions on QoL in MD patients remains inadequately explored.

**Objective:**

To investigate the prevalence of comorbidities in patients with MD and analyze their impact on patients’ QoL.

**Methods:**

A cross-sectional analysis was conducted on 473 MD patients who received treatment in our hospital. Demographic data, comorbidity information, psychological symptoms (assessed by Hospital Anxiety and Depression Scale, HAD) and QoL (evaluated by Ménière’s disease Quality of Life Questionnaire, MDoQ) were collected. Spearman correlation analysis was used to explore the relationship between comorbidities and QoL scores, and independent-samples t-test was applied for intergroup comparison. A multiple linear regression analysis was conducted to identify comorbidities influencing MDoQ.

**Results:**

The study included 328 females (69.3%) and 145 males (30.7%) with a mean age of 45.6 ± 14.5 years and an average disease duration of 4.5 ± 2.0 years. The most common comorbidities were hypertension (49.0%), sleep disorders (36.6%), and diabetes mellitus (28.8%). Clear anxiety symptoms were observed in 59.2% of subjects, whereas distinct depressive symptoms were detected in 53.9%. The mean MDoQ score was 43.62 ± 12.50 (range: 25–76). Correlation analysis revealed that hypertension (*r* = −0.521, *p* < 0.001), diabetes mellitus (*r* = −0.459, *p* < 0.001), sleep disorders (*r* = −0.447, *p* < 0.001), anxiety (*r* = −0.438, *p* < 0.001) and depression (*r* = −0.391, *p* < 0.001) were strongly negatively correlated with MDoQ scores. Multiple linear regression analysis showed that hypertension, diabetes, sleep disorder, migraine, thyroid disease and anxiety symptoms were significant factors affecting the QoL of patients with MD (*F* = 114.18, *p* < 0.001).

**Conclusion:**

Comorbidities are highly prevalent in MD patients, with hypertension, sleep disorders and diabetes mellitus being the most common. These comorbidities, together with the high incidence of anxiety and depression, significantly impair patients’ QoL. Comprehensive management of MD should include systematic screening, early intervention for common comorbidities and psychological support to improve patients’ clinical outcomes and QoL.

## Introduction

Ménière’s disease (MD) is an idiopathic inner ear disorder characterized by recurrent episodes of vertigo, accompanied by fluctuating, progressive sensorineural hearing loss, tinnitus, and a sensation of fullness in the ear (1, 2). MD most commonly affects individuals aged 40–60, with a higher prevalence in women than men (3). Current literature on the incidence and prevalence of MD reported significant variations across different countries, regions and ethnic groups (4–6). In 2020, the American Academy of Otolaryngology–Head and Neck Surgery reported the prevalence of MD to be (50–200) per 100,000 (7). The disease primarily affects middle-aged adults and has a profound impact on patients’ daily activities, occupational function, and psychological well-being (8). Notably, impaired QoL and concurrent psychiatric symptoms represent critical adverse outcomes in patients with vertigo caused by various vestibular disorders. A recent cross-sectional study covering multiple vestibular disorders further confirmed that patients with MD experience both decline in QoL and depressive symptoms (9). With the deepening of clinical research, it has been recognized that MD is not an isolated otological disease but is often associated with a variety of systemic comorbidities (10, 11).

Research has revealed that MD clinically linked to a variety of other conditions, such as migraine (12), thyroid disease (13), anxiety, depression (14), and sleep disturbances (15). Several studies have proposed the association between MD and migraine (12, 16), and some studies have found that migraine is described as a common comorbid disease of MD (17). MD can also present with headaches, which may complicate the differential diagnosis between MD and migraine. A prospective study reported that the prevalence of migraine in 78 MD patients was higher than that in 78 matched patients (56% vs. 25%) (16). Research has identified associations between thyroid disorders such as goiter, hypothyroidism and hyperthyroidism, and MD (13). A meta-analysis regarding thyroid disorders and MD indicated that thyroid hormone treatment not only improved hypothyroidism symptoms but also MD (18). Several studies have demonstrated that some patients with MD were complicated with a certain degree of anxiety and depression, and affected the patient’s sleep (14, 15). Improving the patient’s sleep and anti-anxiety or depression treatment can also improve the patient’s manifestations of MD (19).

An in-depth understanding of the prevalence of comorbidities in MD patients and their correlation with quality of life (QoL) is of great significance for optimizing clinical management strategie. This study aimed to explore the prevalence of common comorbidities in a large cohort of Chinese MD patients, evaluate the psychological status and QoL of the patients, and analyze the impact of comorbidities on QoL, so as to provide evidence-based reference for the comprehensive diagnosis and treatment of MD.

## Methods

### Ethics approval and consent to participate

This cross-sectional study was approved by the Ethics Committee of Xiaolan People’s Hospital of Zhongshan (approval no. ZSXL-LL2019-046) on January 17, 2019, in compliance with the ethical principles outlined in the 1964 Declaration of Helsinki. Informed consent was obtained in accordance with the requirements for informed consent.

### Study population and participant selection

The patients with definite MD in Xiaolan People’s Hospital of ZhongShan from January 2020 to June 2025 were collected. Detailed diagnostic criteria for definite MD were defined as (20): (1) Two or more spontaneous episodes of vertigo, each lasting 20 min to 12 h. (2) Audiometrically documented low to medium frequency sensorineural hearing loss in one ear, defining the affected ear on at least one occasion before, during or after one of the episodes of vertigo. (3) Fluctuating aural symptoms (hearing, tinnitus or fullness) in the affected ear. (4) Not better accounted for by another vestibular diagnosis.

Inclusion criteria: (1) Complete clinical data available and meeting above definite MD diagnostic criteria based on ICVD guideline and MD (2017) (20, 21). (2) Age range from 18 to 80 years. (3) Patients were both willing and able to cooperate in completing the relevant questionnaires. Exclusion criteria: (1) Complicated with definite inner ear diseases, such as labyrinthitis, acoustic neuroma and primary sudden sensorineural hearing loss (SSNHL). (2) Previously diagnosed independent vestibular diseases including benign paroxysmal positional vertigo (BPPV), vestibular neuritis and vestibular paroxysmia. (3) Patients with pre-existing primary psychiatric disorders or chronic organic sleep disorders diagnosed by specialist physicians were excluded.

### Comorbidities

We collected baseline demographic data, medical history, laboratory test results, and other information from patients. The definition of relevant medical history is as follows:

History of hypertension: Hypertension was diagnosed based on the 2017 American College of Cardiology/American Heart Association (ACC/AHA) Guideline for the Prevention, Detection, Evaluation, and Management of High Blood Pressure in Adults (22). The diagnostic criterion was repeated blood pressure ≥140/90 mmHg (1 mmHg = 0.133 kPa) or persistent use of antihypertensive drugs, and all cases were diagnosed by cardiologists.

History of diabetes: Diabetes mellitus was diagnosed in accordance with the Standards of Medical Care in Diabetes—2019 issued by the American Diabetes Association (ADA) (23). Patients with two separate 2-h oral glucose tolerance test results ≥11.1 mmol/L or long-term use of hypoglycemic agents were diagnosed with diabetes. All diagnoses were confirmed by endocrinologists.

History of sleep disorder: The diagnosis of sleep disorders was mainly based on self-assessment by patients using the internationally recognized Athens Insomnia Scale (AIS) (24), with each item scoring 0–3 points and a total score of 0–24 points. The score greater than 6 was considered a sleep disorder, and the higher the score, the more severe the insomnia.

History of thyroid disease: Diagnosed by endocrinologists, including goiter, hypothyroidism, thyroiditis, hyperthyroidism, and autoimmune thyroiditis.

History of migraine: Migraine was diagnosed strictly by neurologists in accordance with the International Classification of Headaches (Third Edition) (25).

### Hospital Anxiety and Depression Scale (HAD)

We used the Hospital Anxiety and Depression Scale (HAD) to assess patients’ anxiety and depression levels. The HAD scale has good reliability and validity for general hospital inpatients, and its Chinese version has been well validated in Chinese populations, supporting its application in otological patients (26, 27). This scale comprises two sections for anxiety and depression, using a 4-point scale (0–3 points) with 14 items. Items 1, 3, 5, 7, 9, 11, and 13 assess anxiety factors, while items 2, 4, 6, 8, 10, 12, and 14 assess depression factors. The criteria for interpretation are as follows: A total score of 0–7 indicates no depression or anxiety. A total score of 8–10 indicates possible or “borderline” depression or anxiety. A total score of 11–20 indicates possible significant depression or anxiety.

### Quality of life assessment

Ménière’s Disease Quality of Life Questionnaire (MDoQ) was used to evaluate the QoL of MD patients (28, 29). The Chinese translated MDoQ has been verified for reliability and validity in Chinese MD cohorts in prior published researches (30). The questionnaire contains 19 items covering 4 domains: physical function, emotional state, social function, and disease-specific symptoms. The response options for each question range from ‘Severe’ (0 points) to ‘None’ (4 points), with a total score of 76 points. Higher scores indicate better QoL.

### Statistical analysis

Statistical analysis was performed using IBM SPSS 26.0 statistical software. The normality of continuous variable distribution was verified via Shapiro–Wilk test prior to statistical testing. Quantitative data (age, disease duration, MDoQ score, HAD score) were expressed as mean ± standard deviation (Mean ± SD) and range, and categorical data (sex, comorbidities, smoking, drinking, anxiety/depression status) were expressed as *n* (%). Independent-samples t-test was used for the comparison of MDoQ scores between patients with and without key comorbidities. Spearman correlation analysis was used to explore the linear correlation between comorbidities and MDoQ scores. One-way ANOVA was used for the comparison of MDoQ scores among different anxiety or depression grades. A multiple linear regression analysis was conducted to identify factors influencing the QoL of patients with MD. *p* < 0.05 was considered statistically significant.

## Results

### Demographic and baseline characteristics

**Figure 1:**
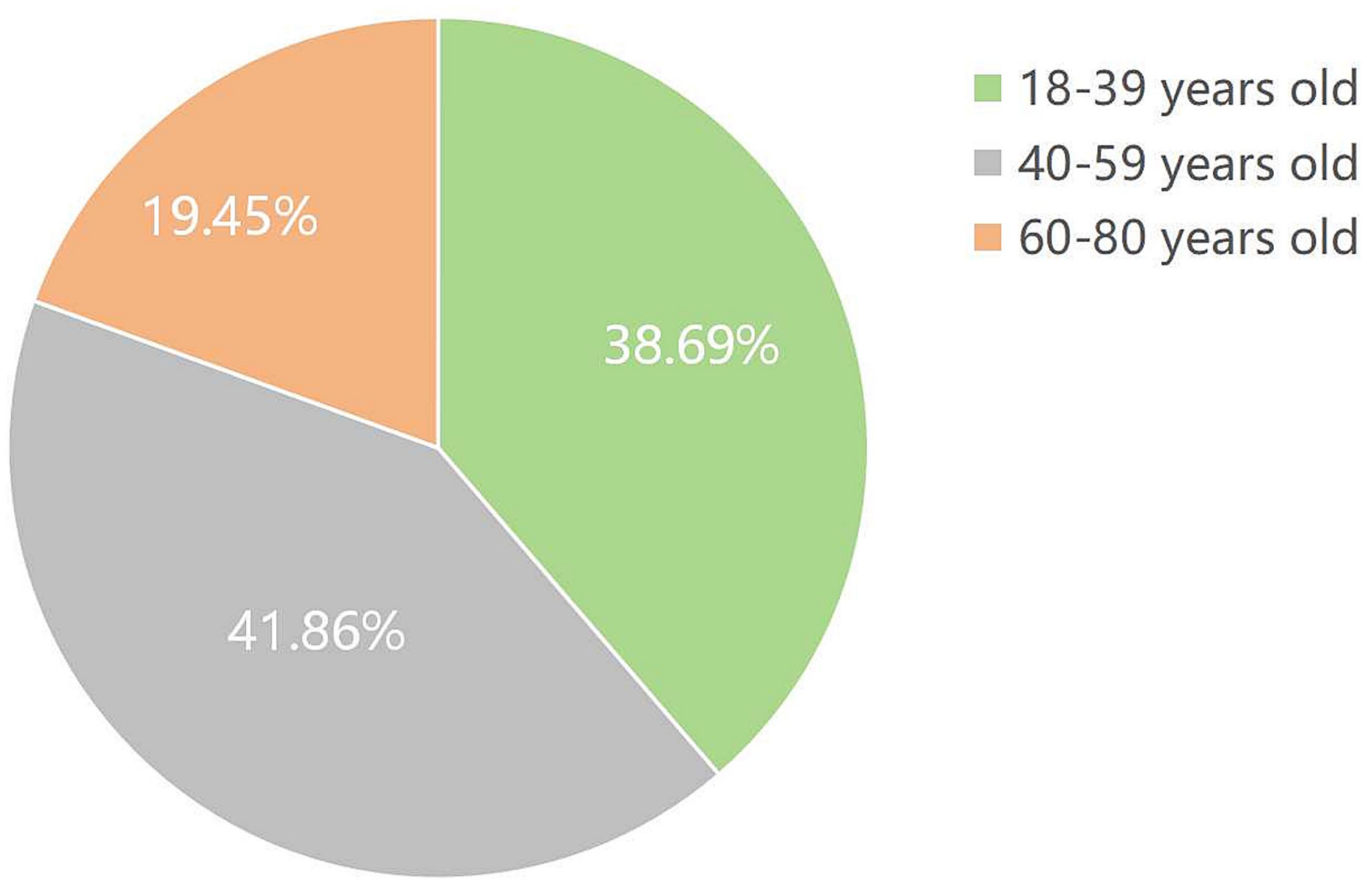
Proportion of Ménière’s disease patients categorized by age group. Pie chart illustrating the age distribution of patients with Meniere’s disease. The cohort was stratified into three age groups: 18–39 years old (green, 38.69%), 40–59 years old (gray, 41.86%), and 60–80 years old (orange, 19.45%).

A total of 473 MD patients were included in the study, including 328 females (69.3%) and 145 males (30.7%), with a female predominance. The mean age of the patients was 45.6 ± 14.5 years, ranging from 18 to 80 years. According to age stratification: 183 patients (38.69%) were 18 ~ 39 years old, 198 patients (41.86%) were 40 ~ 59 years old, and 92 patients (19.45%) were over 60 years old (≤80 years) (Figure 1). The average disease duration was 4.5 ± 2.0 years, ranging from 1 to 9 years. In terms of lifestyle habits, 121 patients (25.6%) had a smoking history and 88 patients (18.6%) had an alcohol consumption history.

### Prevalence of comorbidities and quality of life (MDoQ score)

**Figure 2:**
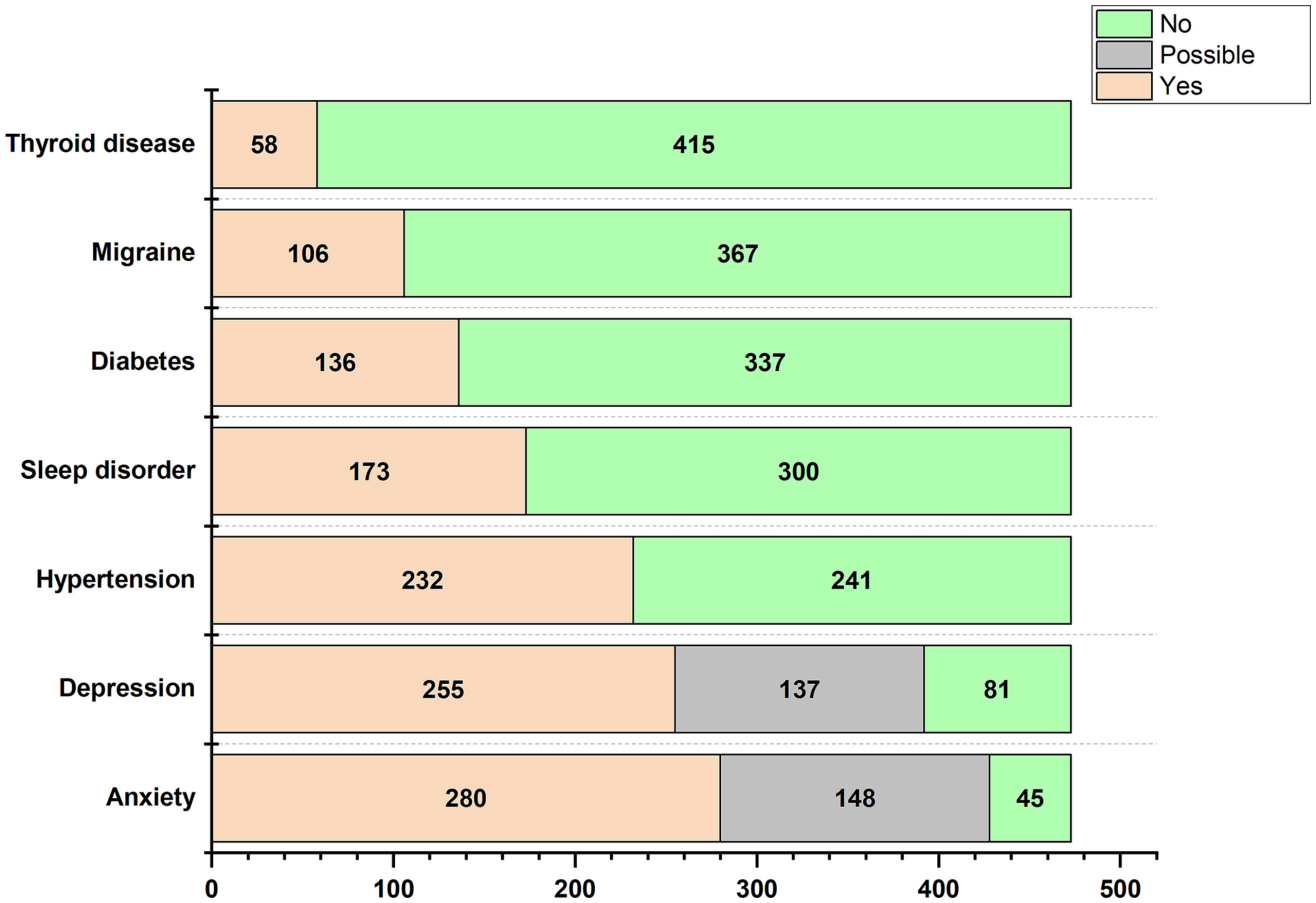
Stacked bar chart showing the prevalence of comorbid conditions in patients with Ménière’s disease. A stacked horizontal bar chart illustrating the distribution of comorbid conditions, categorized as “Yes” (diagnosed, light orange), “Possible” (suspected, gray), and “No” (absent, light green). The number of patients in each category is displayed within the corresponding bar segment.

Comorbidities were highly prevalent in MD patients, with hypertension being the most common (49.0%, 232/473), followed by sleep disorders (36.6%, 173/473) and diabetes mellitus (28.8%, 136/473). The prevalence of other comorbidities was as follows: migraine (22.4%, 106/473), and thyroid diseases (12.3%, 58/473). 59.2% of participants exhibited marked symptoms of anxiety, while 53.9% exhibited marked symptoms of depression (Figure 2).

**Figure 3:**
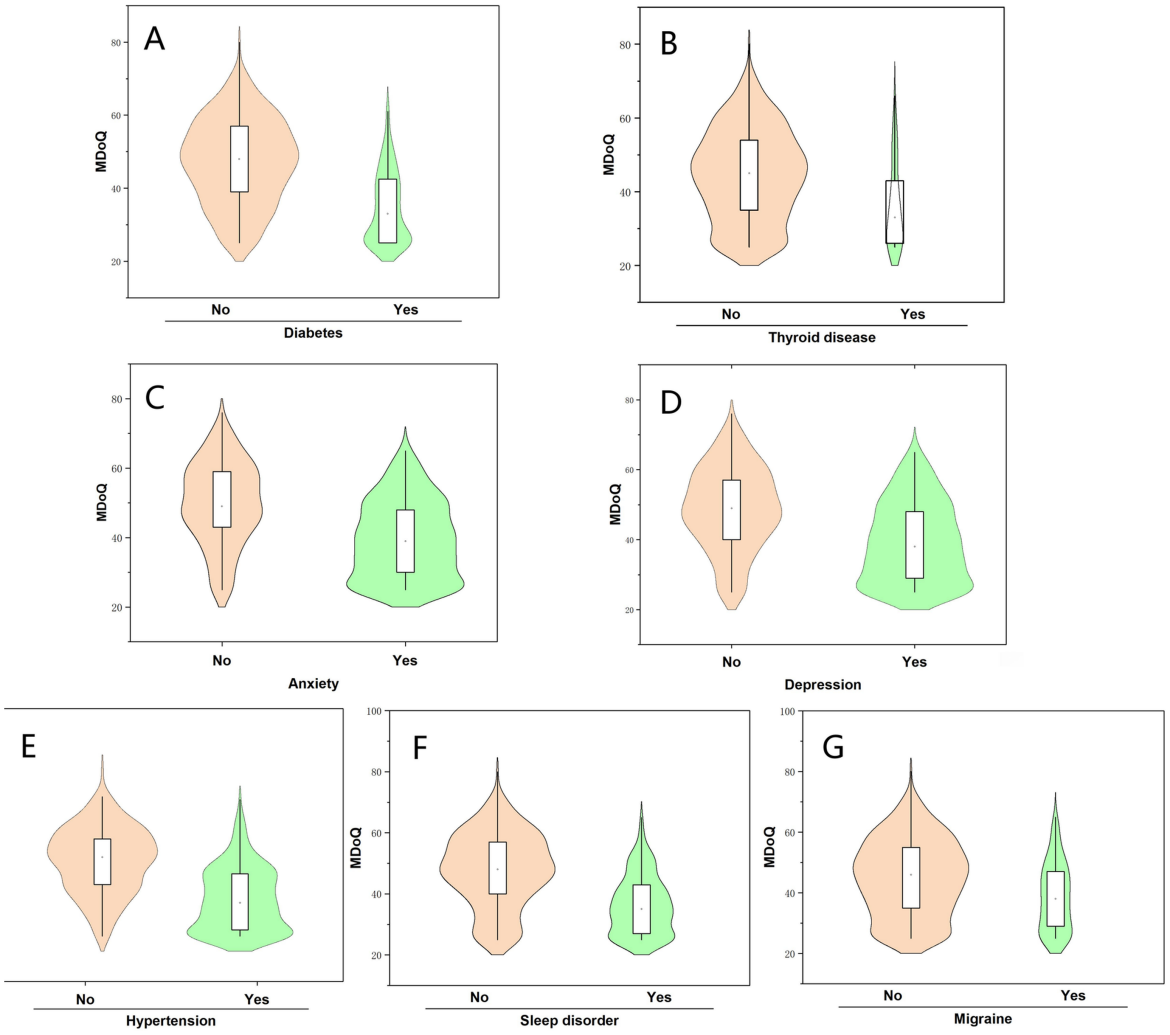
Violin plots with superimposed box plots showing the distribution of MDoQ scores in patients with Ménière’s disease stratified by comorbid status. Violin plots with embedded boxplots illustrate the distribution of MDoQ scores in patients with (green) and without (peach) each comorbidity: **(A)** Diabetes mellitus, **(B)** Thyroid disease, **(C)** Anxiety, **(D)** Depression, **(E)** Hypertension, **(F)** Sleep disorder, and **(G)** Migraine. The central box in each plot represents the interquartile range (IQR, 25th–75th percentiles), the horizontal line inside the box denotes the median, and the whiskers extend to the minimum and maximum non-outlier values. The shape of the violin reflects the kernel density distribution of scores, highlighting differences in score spread and central tendency between groups.

The mean MDoQ score of the 473 MD patients was 43.62 ± 12.50, with a median of 44.0 and a range of 25 ~ 76. A two-sample t-test was conducted to determine whether there was a difference in MDoQ scores among patients with a particular comorbidity. The results showed that patients with sleep disorders, diabetes, anxiety and depression had significantly lower MDoQ scores than those without these conditions, and the difference was statistically significant (*p* < 0.05) (Table 1; Figure 3).

**Table 1:** Baseline characteristics in patients with Ménière’s disease: a comparative analysis according to comorbidity status.

Weighted number (*n*, %)	Total	Sex (F *n*, %)	Age (Mean ± SD)	Duration (years)	Smoking (*n*, %)	Alcohol abuse (*n*, %)	MDoQ (Mean ± SD)
Total	473	69.3%	45.6 ± 14.5	4.5 ± 2.0	121 (25.6%)	88 (18.6%)	43.6 ± 12.5
Hypertension (*n*,%)							
Yes	232 (49%)	165 (68.4%)	47.5 ± 15.9*	4.7 ± 2.0*	64 (27.5%)	40 (17.2%)	37.0 ± 10.1
No	241 (51%)	163 (70.2%)	43.7 ± 12.7*	4.3 ± 1.9*	57 (23.5%)	48 (19.9%)	49.9 ± 10.9
Diabetes (*n*, %)							
Yes	136 (28.8%)	100 (73.5%)	46.4 ± 15.7	4.6 ± 2.0	33 (24.2%)	23 (16.9%)	34.7 ± 9.6*
No	337 (71.2%)	228 (67.6%)	45.3 ± 15.9	4.5 ± 2.0	88 (26.1%)	65 (19.2%)	47.2 ± 11.7*
Sleep disorder (*n*, %)							
Yes	173 (36.6%)	138 (79.7%)*	48.3 ± 14.5	4.6 ± 1.9	39 (22.5%)	22 (12.7%)*	36.4 ± 9.7*
No	300 (63.4%)	190 (63.3%)*	44.1 ± 14.2	4.4 ± 2.0	82 (27.3%)	66 (22.0%)*	47.8 ± 12.1*
Thyroid disease (*n*, %)							
Yes	58 (12.3%)	46 (79.3%)	51.8 ± 14.4	5.38 ± 2.0	16 (27.5%)	10 (17.2%)	36.5 ± 11.5
No	415 (87.7%)	282 (67.9%)	44.7 ± 14.3	4.38 ± 1.9	105 (25.3%)	78 (18.7%)	44.6 ± 12.3
Migraine (*n*,%)							
Yes	106 (22.4%)	80 (75.4%)	52.6 ± 15.5	5.3 ± 1.9	24 (22.6)	15 (14.1%)	38.8 ± 11.0
No	367 (77.6%)	248 (67.5%)	43.6 ± 13.5	4.3 ± 1.9	97 (26.4%)	73 (19.8%)	45.0 ± 12.6
Anxiety (*n*, %)							
Yes	280 (59.2%)	210 (75%)*	47.2 ± 15.0*	4.7 ± 2.0*	65 (23.2%)	43 (15.3%)	39.4 ± 11.1*
Possible	148 (31.3%)	91 (61.4%)*	43.0 ± 13.0*	4.3 ± 1.9*	43 (29.0%)	33 (22.2%)	47.9 ± 11.5*
No	45 (9.5%)	27 (60%)*	44.1 ± 14.7*	4.0 ± 1.9*	13 (28.8%)	12 (26.6%)	55.7 ± 11.5*
Depression (*n*, %)							
Yes	255 (53.9%)	186 (72.9%)	47.5 ± 15.1*	4.7 ± 2.0*	62 (24.3%)	43 (16.9%)	38.5 ± 11.2*
Possible	137 (28.9%)	87 (63.5%)	43.7 ± 13.8*	4.4 ± 2.0*	36 (26.3%)	27 (19.7%)	46.8 ± 11.4*
No	81 (17.1%)	55 (67.9%)	43.9 ± 14.2*	4.1 ± 1.9*	23 (28.4%)	18 (22.2%)	53.2 ± 11.3*

F = Female; *Indicates statistical differences in comparison (*p* < 0.05).

## Correlation analysis

Spearman correlation analysis showed that most comorbidities were negatively correlated with MDoQ scores. Among them, hypertension (*r* = −0.521, *p* < 0.001), diabetes mellitus (*r* = −0.459, *p* < 0.001), sleep disorders (*r* = −0.447, *p* < 0.001) symptoms of anxiety (*r* = −0.438, *p* < 0.001) and depression (*r* = −0.391, *p* < 0.001) were strongly negatively correlated with MDoQ scores, indicating that patients with these comorbidities had significantly lower QoL (Table 2).

**Table 2:** Correlation analysis between MDoQ scores with comorbidities in patients with Ménière’s disease.

Comorbidity	*r*	*P*
Hypertension	−0.521	<0.001
Diabetes	−0.459	<0.001
Sleep disorder	−0.447	<0.001
Thyroid disease	−0.214	<0.001
Migraine	−0.208	<0.001
Anxiety	−0.438	<0.001
Depression	−0.391	<0.001

### Multiple linear regression analysis of factors affecting quality of life

The overall regression model was statistically significant (*F* = 114.18, *p* < 0.001), with an *R*^2^ of 0.703 and an adjusted *R*^2^ of 0.696, indicating that the included comorbidities collectively accounted for 69.6% of the variance in QoL scores. Several comorbidities were independently and significantly associated with poorer QoL (all *p* < 0.001). Hypertension (*B* = −10.11, *β* = −0.30), diabetes (*B* = −10.60, *β* = −0.31), and sleep disorders (*B* = −9.14, *β* = −0.26) exhibited the strongest negative associations. Thyroid disease (*B* = −5.19, *β* = −0.12), migraine (*B* = −3.64, *β* = −0.09), and anxiety (*B* = −4.30, *β* = −0.11) were also significantly linked to reduced QoL. In contrast, depression showed no significant association with QoL in this cohort (*B* = −0.38, *β* = −0.01, *p* = 0.690) (Table 3).

**Table 3:** Multiple linear regression analysis of factors affecting the quality of life of patients with Ménière’s disease.

Comorbidity	*B*	SE	Beta	*t*	*p*
Hypertension	−10.11	0.66	−0.30	−15.23	<0.001
Diabetes	−10.60	0.73	−0.31	−14.61	<0.001
Sleep disorder	−9.14	0.75	−0.26	−12.20	<0.001
Thyroid disease	−5.19	0.99	−0.12	−5.23	<0.001
Migraine	−3.64	0.81	−0.09	−4.53	<0.001
Anxiety	−4.30	1.11	−0.11	−3.87	<0.001
Depression	−0.38	0.95	−0.01	−0.04	0.690

Regression coefficients (*B*), standard errors (SE), standardized coefficients (Beta), *t*-values, and *p*-values are presented for each comorbidity. The model was adjusted for age, disease duration, and sex. *R*^2^ = 0.703, adjusted *R*^2^ = 0.696, *F* = 114.18, *p* < 0.001.

## Discussion

This single-center cross-sectional study enrolled 473 patients diagnosed with definite MD. Hypertension, sleep disorders and diabetes mellitus were the three most prevalent comorbidities. Clinically significant anxiety and depression were present in 59.2 and 53.9% of the cohort. Hypertension, diabetes mellitus, sleep disorders, migraine, thyroid disease and anxiety were confirmed as independent risk factors for impaired QoL, whereas depression showed no independent association. Overall, comorbidities are highly prevalent among Chinese MD patients and closely linked to reduced QoL. MD is a chronic inner ear disorder that imposes a substantial burden on patients’ daily function and psychological well-being (10, 31, 32). Emerging evidence has recognized MD as a systemic condition rather than an isolated otological problem, with a high rate of concomitant comorbidities that may synergistically worsen disease severity and QoL (2, 10, 14, 15). These findings underscore the necessity of comprehensive, multidisciplinary care integrating somatic comorbidity screening and psychological support into routine MD management.

The high prevalence of hypertension observed in our cohort is consistent with population-based evidence linking cardiovascular and metabolic conditions to MD. A large-scale epidemiologic study by Kim reported significant associations between MD and hypertension (10), supporting the hypothesis that vascular dysregulation contributes to inner ear homeostasis disruption and endolymphatic hydrops formation. Elevated blood sodium levels are closely involved in the interaction between hypertension and inner ear dysfunction. Accordingly, salt restriction is regarded as a core dietary intervention for MD patients (33). Our data extend these observations by revealing a strong negative correlation between hypertension and MDoQ scores, suggesting that uncontrolled blood pressure directly compromises physical, emotional, and social QoL domains in affected individuals. Similarly, diabetes mellitus, which is a chronic metabolic condition affecting cochlear, vestibular, and balance system functions, was highly prevalent and negatively associated with QoL (34, 35). Chronic hyperglycemia may impair microvascular perfusion of the inner ear, exacerbate vestibular dysfunction, and amplify symptom burden (36). Collectively, these metabolic–vascular comorbidities appear to act as critical effect modifiers in MD, justifying systematic screening and targeted cardiometabolic control in clinical protocols (37, 38).

Sleep disorders represented another highly prevalent comorbidity and a powerful predictor of reduced QoL. Recent investigations have highlighted reciprocal interactions between vestibular dysfunction and sleep architecture, with vertigo attacks disrupting sleep continuity and sleep deprivation lowering vestibular compensation and increasing dizziness sensitivity (19, 39, 40). A 2025 study by Petry et al. similarly documented a high prevalence of sleep disturbances in MD and emphasized the bidirectional interplay between peripheral vestibular function and central sleep regulation (15). Our results reinforce that sleep impairment is not merely a secondary consequence of MD but an independent comorbid condition requiring active intervention to improve QoL.

A recent systematic review and meta-analysis by Yeo et al. (14) indicated a consistent and significant associations between MD and depression/anxiety, with the estimated prevalence of anxiety and depression reaching 34 and 23%, respectively, across 35 independent cohorts. Chronic vertigo, fluctuating sensorineural hearing loss, and tinnitus can cause persistent uncertainty and distress, promoting maladaptive cognitive emotional responses that further reduce QoL (2, 41, 42). Notably, the temporal and causal relationships between psychological symptoms and MD remain unclear. It remains unclear whether anxiety and depression are pre-existing mental health conditions that predispose individuals to MD or exacerbate its severity, or whether they are psychological consequences of this chronic condition (43). MD’s recurrent, unpredictable vertigo attacks, progressive hearing loss, and persistent tinnitus caused profound uncertainty, functional impairment, and social withdrawal, all of which are known risk factors for secondary anxiety and depression. Conversely, pre-existing anxiety may heighten physiological stress responses, potentially worsening inner ear inflammation and vestibular instability (44). This study found that anxiety (rather than depression) is an independent predictor of QoL, suggesting that anxiety may be more directly tied to MD-related functional impairment and symptom burden. Anxiety may arise acutely in response to vertigo episodes and persistent vestibular uncertainty, whereas depression may develop more gradually as a result of chronic disability, social isolation and reduced QoL. Importantly, depression’s association with QoL was fully explained by anxiety and comorbidities in our adjusted model, indicating that depression may be a downstream consequence rather than an independent risk factor in this cohort.

We also detected meaningful comorbidities with migraine and thyroid disorders, both of which independently predicted lower QoL. Multiple studies have validated the clinical link between MD and migraine, possibly sharing underlying mechanisms of trigeminal vestibular sensitization and ion channel dysfunction (16–18, 45). Likewise, thyroid dysfunction including hypothyroidism, hyperthyroidism, and autoimmune thyroiditis, has been associated with MD in many studies, suggesting that hormonal imbalance may disrupt inner ear fluid regulation (46–49). Our findings consolidate these associations and demonstrate that both migraine and thyroid disease contribute additively to QoL impairment.

Coexisting multiple comorbidities impair patients’ QoL via multiple mechanisms. First, hypertension, diabetes mellitus, thyroid disorders and migraine can directly aggravate inner ear lesions and vestibular symptoms of MD, exacerbating physical discomfort. Second, overlapping symptoms and long-term disease-related distress further elevate psychological stress. Furthermore, deteriorated physical symptoms and negative emotions form a vicious cycle, which limits patients’ daily activities and social engagement. Collectively, these factors account for the poorer QoL observed in MD patients with comorbidities.

### Limitations and future directions

Several limitations should be acknowledged. First, the single-center design carries inherent selection and information biases, and generalizability to other ethnic or geographic cohorts requires confirmation. Secondly, although cross-sectional studies can reveal correlations, it cannot elucidate the causal relationship or longitudinal trajectory between comorbidity and QoL, and it remains unclear whether anxiety and depression are pre-existing comorbidities or psychological consequences of MD. Third, we lacked a healthy control group or a control group with other vestibular disorders, limiting direct comparative analyses. Future research should employ prospective, multicenter designs with long-term follow-up to clarify causal mechanisms and evaluate whether targeted comorbidity intervention improves vertigo control, hearing preservation, and patient-reported outcomes.

## Conclusion

In conclusion, Chinese patients with MD not only carry a high burden of comorbidities such as hypertension, diabetes, sleep disorders, migraines and thyroid disorders, but also suffer from psychological distress such as anxiety and depression. These conditions are independently and negatively associated with QoL and act in a cumulative manner. Comprehensive clinical care for MD should therefore incorporate systematic screening for cardiometabolic, sleep-related, migraine, thyroid, and psychological symptoms, followed by personalized multidisciplinary interventions. Such integrated strategies are expected to reduce symptom severity improve functional capacity, and ultimately enhance long-term QoL in this vulnerable patient population. Multidisciplinary collaboration and individualized treatment plans are key to improving both clinical outcomes and QoL in MD patients.

## Data Availability

The raw data supporting the conclusions of this article will be made available by the authors, without undue reservation.
